# Shiitake mushroom‐induced flagellate dermatitis: The first case reported in Nepal

**DOI:** 10.1002/ccr3.9228

**Published:** 2024-07-28

**Authors:** Elisha Shrestha, Manisha Singh Basukala, Aditi Mishra, Namrata Pradhan, Anupa Sharma, Ashish Tamang

**Affiliations:** ^1^ Department of Dermatology, Venereology and Leprosy, Kathmandu University School of Medical Sciences Dhulikhel Hospital Dhulikhel Nepal; ^2^ Kathmandu University School of Medical Sciences, Dhulikhel Hospital Dhulikhel Nepal

**Keywords:** dermatitis, erythema, fungi, lentinan, shiitake mushrooms

## Abstract

Shiitake mushroom‐induced flagellate dermatitis is a rare but important condition to consider in patients presenting with pruritic skin lesions following mushroom ingestion, especially in regions where such cases are uncommon. Early recognition and appropriate management with oral and topical steroids are crucial for effective symptom resolution and preventing complications. Clinicians should be aware of the characteristic rash and the temporal relationship between mushroom consumption and rash onset. Educating patients about the risks associated with consuming raw or undercooked shiitake mushrooms is essential to prevent recurrence.

## 1 INTRODUCTION

Shiitake mushroom‐induced flagellate dermatitis, also known as flagellate erythema, is an intriguing rash associated with consuming raw or undercooked shiitake mushrooms (Lentinula edodes).^1^ While these mushrooms are widely appreciated for their culinary and potential health benefits, they can occasionally trigger an immune‐mediated skin reaction in susceptible individuals. The characteristic rash manifests as intensely pruritic linear streaks, resembling whiplash marks, and typically appears within 24 h to 5 days after mushroom ingestion.^2^

The pathogenesis of shiitake flagellate dermatoses centers around a thermolabile polysaccharide called lentinan, which is present in shiitake mushrooms. Lentinan activates interleukin‐1 secretion, leading to vasodilation, hemorrhage, and the distinctive rash.^3^ Notably, this flagellate dermatosis is not observed when mushrooms are thoroughly cooked at temperatures exceeding 145°C.^4^ As mushroom consumption continues to rise globally, recognizing this etiology becomes crucial, preventing diagnostic challenges and the recurrence of this characteristic rash.

This case report is particularly significant as it documents the first known instance of shiitake mushroom‐induced dermatitis in Nepal, highlighting the need for awareness and consideration of this condition even in regions where it is not typically prevalent. Given the growing popularity of shiitake mushrooms in Nepal, there is a significant likelihood of incidence of shiitake dermatitis cases arising.

## 2 CASE HISTORY AND EXAMINATION

**FIGURE 1:**
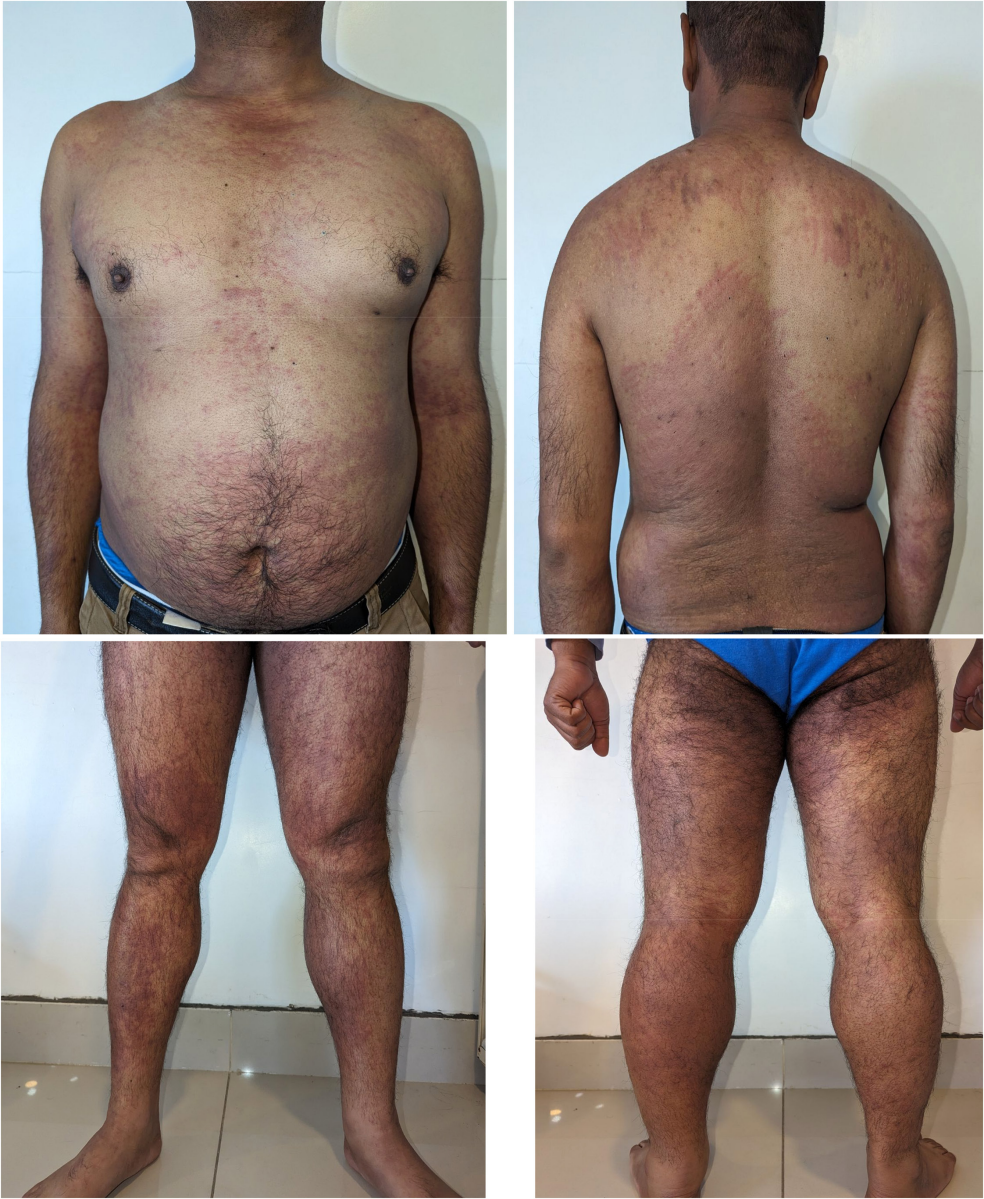
Figure showing multiple grouped linear and oblique erythematous plaques and papules distributed across the shoulders, neck, back, abdomen, and lower limbs of the patient. Additionally, discrete erythematous papules and macules are observed on the chest and upper limbs, presenting as intensely pruritic and erythematous streaks resembling whiplash marks. These lesions are characteristic of shiitake mushroom‐induced flagellate dermatoses.

**FIGURE 2:**
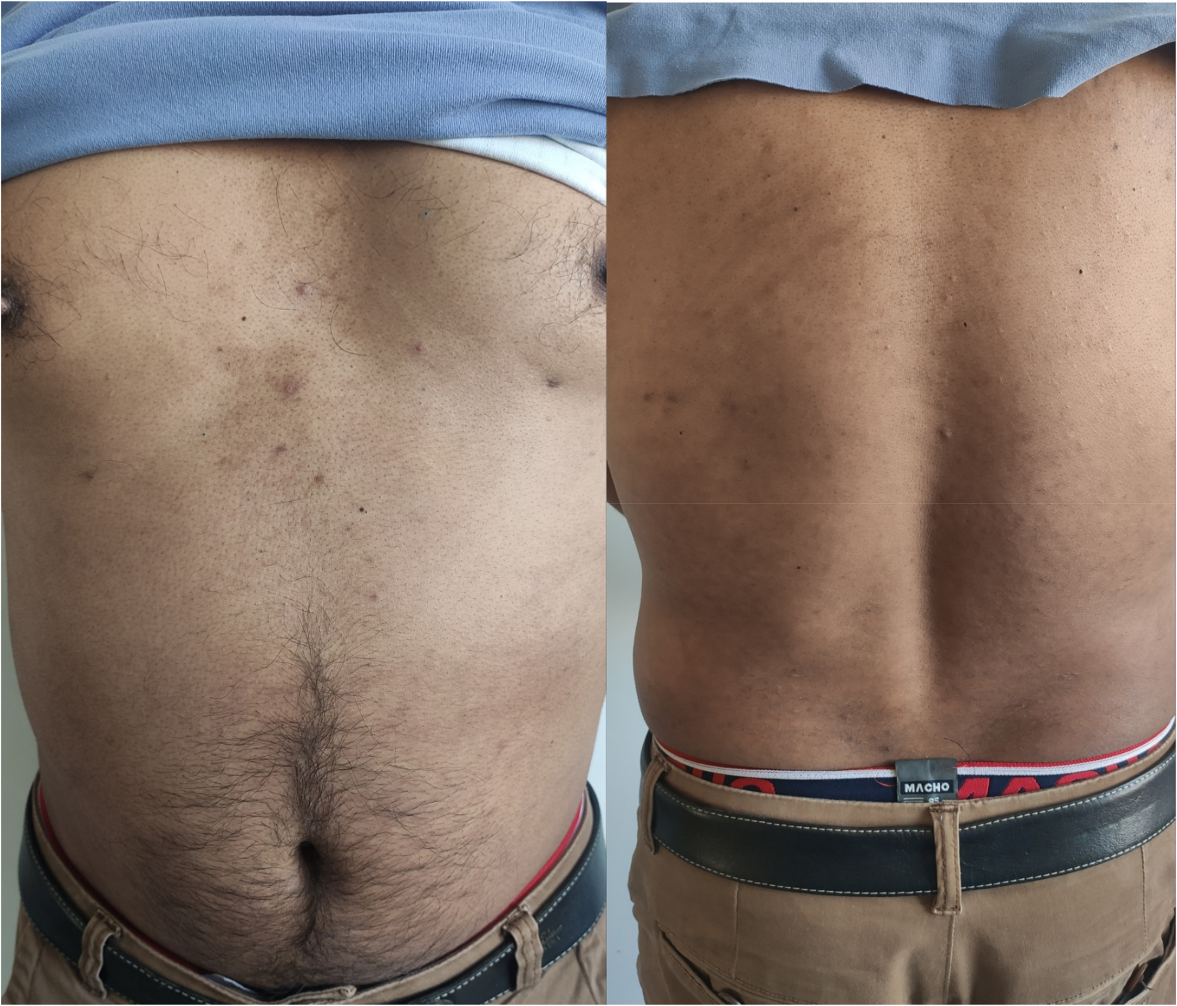
The image shows significant improvement in the patient's skin condition following 7 days of treatment with oral and topical steroids. The previously affected areas, including the chest, back, and upper extremities, exhibit almost complete resolution of pruritus and a marked reduction in erythema. Only mild residual redness remains, indicating an effective response to the therapeutic intervention.

A 38‐year‐old male presented to our dermatology clinic with a chief complaint of itchy linear reddish rashes mostly over the entire body for three days. The rash initially appeared on his lower limbs then gradually progressed to involve chest, back, and upper extremities. Initially, these were a few linear reddish rashes that gradually increased in number which later coalesced and appeared larger. There was no history of use of any soap, shampoo, or detergents or exposure to any plants or other irritants before the onset of the rash. It was not associated with any fluid or pus‐filled lesions. He denied any bug bites. He had no prodromal symptoms and no exposure to any medications before the onset of the rash. The lesions did not slough nor affect his oral mucosa. However, upon further inquiry about his food intake, he revealed that he had consumed special mushrooms fried in oil (Shiitake mushrooms) for 2 days before the rash appeared. He continued consuming them until the day of his presentation. He had never had those mushrooms previously. Physical examination revealed multiple well‐defined linear and oblique erythematous papules and plaques (Figure 1) distributed across his shoulders, neck, back, abdomen and lower limbs. Additionally, discrete erythematous plaques were observed on his chest and upper limbs. A combination of oral steroids (0.5 mg/kg) two times a day for 5 days and topical steroids (cream betamethasone) was administered for 5 days during the initial presentation along with antihistamine (cetirizine 10 mg daily) for 5 days. The patient returned to the clinic for a follow‐up visit after 7 days. Notably, he reported almost full resolution of pruritus (itching), and on examination, there was only mild erythema. The previously affected areas, including the chest, back, and upper extremities, showed significant improvement (Figure 2).

## 3 METHODS

The differential diagnosis included contact dermatitis, atopic dermatitis, drug reactions, and infectious etiologies, with systemic symptoms such as fever and joint pain being notably absent. The diagnosis was primarily clinical, based on the characteristic rash and its temporal relationship with mushroom ingestion. Laboratory investigations, including complete blood count (CBC) and liver function tests (LFTs), were conducted to rule out other systemic involvements. Random blood sugar was also tested, which was normal before introducing steroids.

The patient was instructed to discontinue mushroom consumption immediately. Treatment included oral prednisone 0.5 mg/kg daily for 5 days then tapered to 0.3 mg per kg for the next 5 days and stopped, and topical corticosteroids (clobetasol propionate cream applied twice daily for 1 week), and oral antihistamines (cetirizine 10 mg daily) for 5 days. Follow‐up visits at 3 and 7 days post‐treatment initiation involved clinical examinations to monitor reduction in pruritus and erythema, and to assess any residual effects and steroid‐induced complications like gastritis, higher blood pressure, and increased blood sugar. The effectiveness of the treatment was evaluated through patient‐reported outcomes and clinical observations.

## 4 CONCLUSION AND RESULTS

Clinicians should include shiitake mushroom‐induced dermatitis in their differential diagnosis when evaluating patients with pruritic skin lesions following mushroom ingestion. Awareness of this rare entity enables timely management and improves patient outcomes. In this case, the patient responded well to the treatment, showing almost complete resolution of pruritus and a significant reduction in erythema within 7 days. Educating patients about the risks associated with consuming raw or undercooked shiitake mushrooms is essential to prevent the recurrence of these dermatitis.

## 5 DISCUSSION

Shiitake mushroom‐induced flagellate dermatitis is an intriguing and infrequent dermatological condition. It arises from exposure to lentinan, a bioactive compound found in shiitake mushrooms.^1^ The hallmark of this condition is the development of erythematous skin lesions, which can be distressing for affected individuals.^4^

Shiitake dermatitis was initially documented by Nakamura in 1997 who reported a series of 23 cases that developed similar characteristic linearly arranged erythematous lesions that occurred after consuming this mushroom.^5^ It is the second most consumed mushroom globally. Its cultivation significantly contributes to the world's mushroom industry, producing two million tons of mushrooms annually, with the market expanding. Due to its anticarcinogenic, antihypertensive, and cholesterol‐lowering properties, shiitake mushrooms have been widely globalized on a large scale.^6^

In Japan and China, shiitake mushroom‐induced dermatitis has a higher prevalence due to the dietary habits of the population, which commonly includes raw or undercooked shiitake mushrooms. This condition is relatively well‐documented in these countries.^5^

In Nepal, Shiitake mushroom cultivation has been increasingly adopted in mountainous areas due to the abundant natural resources and suitable altitude.^7^ However, there are very few cases reported in the South Asian population, where shiitake mushrooms are not as commonly consumed.

In a review paper by Nakamura, 51 cases of shiitake dermatitis were observed from 1974 to 1991, following the ingestion of partially cooked raw shiitake mushrooms.^5^ All patients exhibited cutaneous involvement on the trunk, with a decreasing frequency of lesions on the extremities, neck, face, and head. The condition typically resolved within 2 days to 2 weeks, averaging 8.5 days for resolution.^5^

The diagnosis of shiitake mushroom‐induced dermatitis is based on the characteristic history of mushroom consumption and the appearance of typical skin lesions. Additionally, ruling out other potential causes of flagellate dermatitis is essential. Skin lesions resemble those seen in bleomycin‐induced flagellate dermatitis, dermatomyositis Still's disease, and occasionally in HIV patients with hypereosinophilic syndrome. Flagellate dermatitis has also been reported in 1%–2% of oncology patients who have been subjected to chemotherapy like bleomycin^8^, ^9^ but unlike bleomycin‐induced flagellate dermatitis, they lack mucosal lesions or hyperpigmentation.^10^, ^11^ Similar to our case where no mucosal lesions were present. Excluding the presence of fever, lymphadenopathy, and edema, particularly of the face, is crucial, as the possibility of an atypical ‘DRESS’‐like syndrome (Drug Reaction with Eosinophilia and Systemic Symptoms) has been reported.^12^ It is important to note that there are no specific laboratory or histopathological findings associated with this condition.^13^, ^14^

The histological findings include preserved epidermis, papillary edema in the dermis, erythrocyte overflow, and superficial and perivascular infiltrates of mononuclear cells, without vasculitis or pigmentary incontinence which is similar to nonspecific spongiotic dermatitis.^15^, ^16^

Although self‐limiting, early recognition, and appropriate management are essential to alleviate symptoms. Prompt intervention is vital for favorable outcomes. In our case, treatment involved a combination of oral and topical steroids along with antihistamines similar to the approach described in the case report by Camila Nemoto de Mendonça et al.^3^ The patient responded well, with a resolution of the lesions. However, individual responses may vary, and close monitoring is necessary to assess treatment efficacy and manage any adverse effects.^12^

Educating patients about the link between mushroom consumption and flagellate dermatitis is crucial. Advising them to avoid consumption of raw and undercooked shiitake mushrooms or other potential triggers can prevent recurrence.

## AUTHOR CONTRIBUTIONS

**Elisha Shrestha:** Conceptualization; formal analysis; investigation; methodology; project administration; resources; visualization; writing – original draft; writing – review and editing. **Manisha Singh Basukala:** Conceptualization; resources; validation; writing – original draft. **Aditi Mishra:** Conceptualization; resources; supervision; visualization; writing – original draft. **Namrata Pradhan:** Conceptualization; formal analysis; supervision; validation; visualization; writing – original draft. **Anupa Sharma:** Conceptualization; resources; supervision; validation; writing – original draft; writing – review and editing. **Ashish Tamang:** Conceptualization; methodology; project administration; validation; visualization; writing – original draft; writing – review and editing.

## FUNDING INFORMATION

No funding was received.

## CONFLICT OF INTEREST STATEMENT

The authors declare that they have no conflict of interest.

## ETHICS STATEMENT

Ethical approval was not required for this study by local or national guidelines. Written informed consent was obtained from the patient for publication of details of their medical care and any accompanying images.

## CONSENT

Written informed consent was obtained from the patient for the publication of this report following the journal's patient consent policy.

## Data Availability

The authors of this manuscript are prepared to provide supplementary information concerning the case report upon official request. All data generated or analyzed during this study are included in this article. Further inquiries can be directed to the corresponding authors.
